# Multiple-omic data analysis of *Klebsiella pneumoniae* MGH 78578 reveals its transcriptional architecture and regulatory features

**DOI:** 10.1186/1471-2164-13-679

**Published:** 2012-11-29

**Authors:** Joo-Hyun Seo, Jay Sung-Joong Hong, Donghyuk Kim, Byung-Kwan Cho, Tzu-Wen Huang, Shih-Feng Tsai, Bernhard O Palsson, Pep Charusanti

**Affiliations:** 1 Department of Bioengineering, University of California, San Diego, La Jolla, CA 92093, USA; 2 Institute of Molecular and Genomic Medicine, National Health Research Institutes, Miaoli, 350, Taiwan; 3 Current address: Central Research Institute, Samsung Petrochemical, Samsung Advanced Institute of Technology, 6th floor, Building 2, Nongseo-dong, Giheung-gu, Yongin, Gyeonggi-do, 446-712, Republic of Korea; 4 Current address: Department of Biological Sciences, Korea Advanced Institute of Science and Technology, 291 Daehak-ro, Yuseong-gu, Daejeon, 305-701, Korea

**Keywords:** *Klebsiella pneumoniae*, Infectious disease, Transcriptional architecture, Omics data, Systems biology

## Abstract

**Background:**

The increasing number of infections caused by strains of *Klebsiella pneumoniae* that are resistant to multiple antibiotics has developed into a major medical problem worldwide. The development of next-generation sequencing technologies now permits rapid sequencing of many *K. pneumoniae* isolates, but sequence information alone does not provide important structural and operational information for its genome.

**Results:**

Here we take a systems biology approach to annotate the *K. pneumoniae* MGH 78578 genome at the structural and operational levels. Through the acquisition and simultaneous analysis of multiple sample-matched –omics data sets from two growth conditions, we detected 2677, 1227, and 1066 binding sites for RNA polymerase, RpoD, and RpoS, respectively, 3660 RNA polymerase-guided transcript segments, and 3585 transcription start sites throughout the genome. Moreover, analysis of the transcription start site data identified 83 probable leaderless mRNAs, while analysis of unannotated transcripts suggested the presence of 119 putative open reading frames, 15 small RNAs, and 185 antisense transcripts that are not currently annotated.

**Conclusions:**

These findings highlight the strengths of systems biology approaches to the refinement of sequence-based annotations, and to provide new insight into fundamental genome-level biology for this important human pathogen.

## Background

The number of infections caused by pathogenic microorganisms that are resistant to at least one antibiotic has grown at an alarming rate over the past several decades. These multi-drug resistant organisms have reduced the clinical utility of many commonly-used antibiotics, and pan-resistant strains now threaten to render some infectious agents nearly untreatable. The Infectious Diseases Society of America has identified six multi-drug resistant pathogens in particular that pose the gravest threat to human health [1,2], one of which is the Gram-negative bacterium *Klebsiella pneumoniae*. *K. pneumoniae* is a member of the family Enterobacteriaceae, and exhibits close genetic relationship to several genera within this family, especially *Escherichia*. Despite this similarity, many *Klebsiella* species, including *Klebsiella pneumoniae*, possess a thick, extracellular polysaccharide capsule that distinguishes this genus from other enterobacteria. This capsule is thought to be a significant virulence factor that helps to protect the bacterium during infection from phagocytosis [3-5] and antimicrobial peptides [6]. *K. pneumoniae* causes a wide range of diseases worldwide such as pneumonia, urinary tract infections, and surgical wound infections that primarily afflict immunocompromised patients. There are also highly invasive community-acquired pathotypes characterized by bacteremic liver abscesses or endophthalmitis that are particularly endemic in Asia [7-9], especially in Taiwan [10-13], and reports of their occurrence are now emerging in other parts of the world [14-20].

To combat the threat posed by *K. pneumoniae* and other drug-resistant pathogens, the genomes from many clinical and laboratory-derived isolates have been sequenced to investigate the genetic basis of infection-relevant phenotypes such as virulence and antibiotic resistance [21,22]. Several notable discoveries have been made through these sequencing efforts, for example the identification of plasmids that bear New Delhi metallo-β-lactamase 1 (NDM-1), a gene that confers resistance to the last-line carbapenem antibiotics that are used to treat difficult *K. pneumoniae* infections [23]. Other studies have sought to build upon the abundance of sequence data to delineate fundamental operational features of the genome, for instance the identity and binding site locations of major transcription factors, the environmental signals that stimulate transcription of certain genes, the architecture of operons, sub-operons, and transcription units, the existence of small non-coding RNAs, and other elements [24-27]. Such studies often rely on the acquisition and analysis of genome-wide data sets such as chromatin immunoprecipitation combined with microarray hybridization (ChIP-chip), transcriptome profiling, proteomics, and metabolomics, ultimately resulting in a global map of the transcriptional architecture for the bacterium under defined growth conditions. In turn, this map provides a fundamental link between the genotype and phenotype for the organism.

Using ChIP-chip, tiling array, and deep sequencing technology, we report here a delineation of the transcriptional architecture for a pathogenic, multi-drug resistant strain of *Klebsiella pneumoniae* during exponential and stationary phase growth. Key findings include the detection of over 1000 binding sites for RpoD and RpoS, nearly 200 RNA transcript segments that have multiple transcription start sites, and over 80 leaderless mRNAs.

## Results

### Analysis of transcriptional architecture

**Figure 1:**
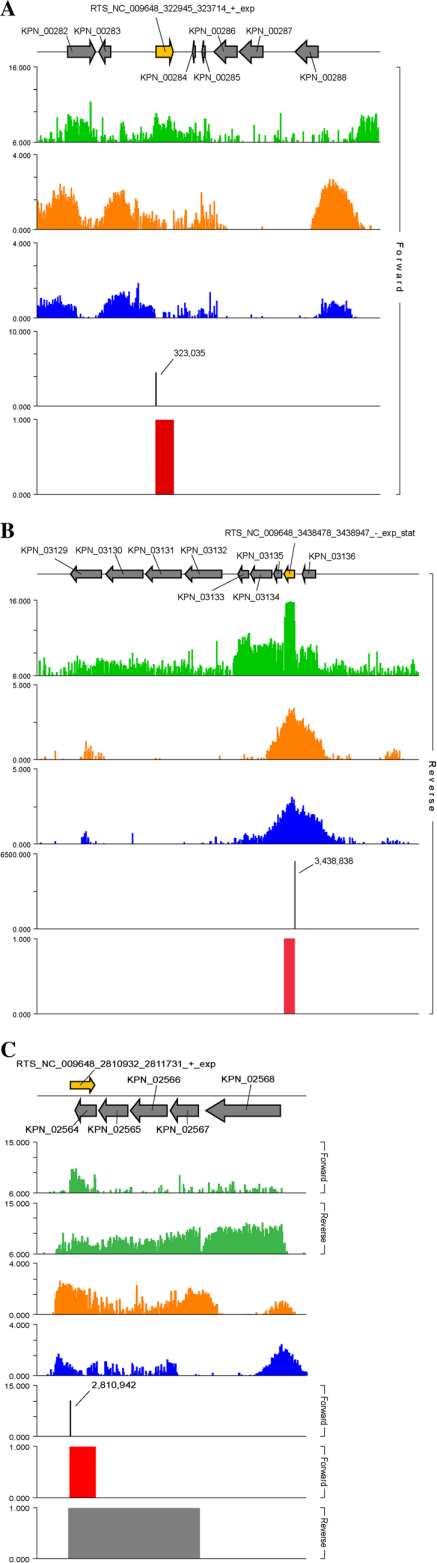
**Experimental elucidation of the transcriptional architecture for *Klebsiella pneumoniae *MGH 78578.** Examples illustrating the identification of a (**A**) new open reading frame from the data sets (HAD hydrolase, family IB, E-value from query vs. best hit by BLASTp search: 7.00E-125); (**B**) an sRNA, CsrB (genomic coordinates: 3,438,478~3,438,829); and (**C**) an antisense transcript against KPN_02564/*yehW* (genomic coordinates: 2810932~2811731). In each panel, the new feature is depicted as a yellow arrow, while gray arrows denote currently annotated genes. Green: Strand-specific transcription data. Orange: RNA polymerase binding data. Blue: RpoD binding data. Black: Transcription start site (TSS) data. Red: newly-determined genomic feature. The name of each RNA-guided transcript segment (RTS) is structured as follows: RTS_genome; locus ID; start site on microarray; stop site on microarray; strand; growth phase. In panel (**C**), the bottom gray rectangle denotes the sense RTS on the forward strand.

To construct the transcriptional architecture of the *K. pneumoniae* MGH 78578 genome, we determined the active coding regions of the genome in both exponential and stationary phase by investigating gene expression, RNAP, RpoD, and RpoS binding sites, and transcription start site (TSS) data collected under these two growth conditions (Figure 1). A particular genomic region was deemed to be transcribed if the gene expression data for that locus was above an estimated baseline value of log_2_(signal) = ~6. Because our microarray lacked negative control probes [24], we based this value on the detected signal from non-coding regions such as intergenic regions. The data were transformed into binary expressed/unexpressed calls based on this threshold, and subsequently used as the basis to determine contiguous transcript segments. RNAP, RpoD, and RpoS binding sites were identified from similar log ratio data sets from triplicate chromatin immunoprecipitation (ChIP) samples using NimbleScan software (width of sliding window: 300 bp). We then calculated the median position of those regions to avoid a skewed position by unwanted noise. We identified RNAP binding sites from ChIP data collected during exponential phase only since the binding sites do not differ appreciably between the exponential and stationary growth phases [24]. In addition, cells were treated with rifampicin to generate a static RNAP binding map [24]. For RpoD or RpoS binding site identification, we prepared a ChIP library from cells harvested in exponential phase and stationary phase, respectively. ChIP samples were then hybridized to a custom-designed tiled microarray to pinpoint the genomic locations where these proteins bound. By comparing RNAP, ChIP-chip data against both RpoD, and RpoS ChIP-chip data and expression data all acquired simultaneously under the same growth conditions, it is possible to segregate what appear to be contiguous transcripts into distinct transcription segments [25]. TSSs were identified using a modified 5^′^-RACE protocol combined with deep sequencing, which only detects mRNAs with triphosphates at the 5^′^ end [25]. The data are summarized in Table 1.

**Table 1:** **Experimentally derived annotation of the *Klebsiella pneumoniae *MGH 78578 genome**

ORFs	5194/411*
tRNAs	86 (86)
rRNAs	25 (25)
sRNAs	15 (1)
RNAP binding sites	2677
RpoD binding sites	1227/143*
RpoS binding sites	1066/82*
TSS	3585/263*
pORF	119/40*
RTSs – total	3660
RTSs – exponential phase	678
RTSs – stationary phase	1003
RTSs – both phases	1979
Antisense transcripts	185/38*
Leaderless mRNAs	83

**Figure 2:**
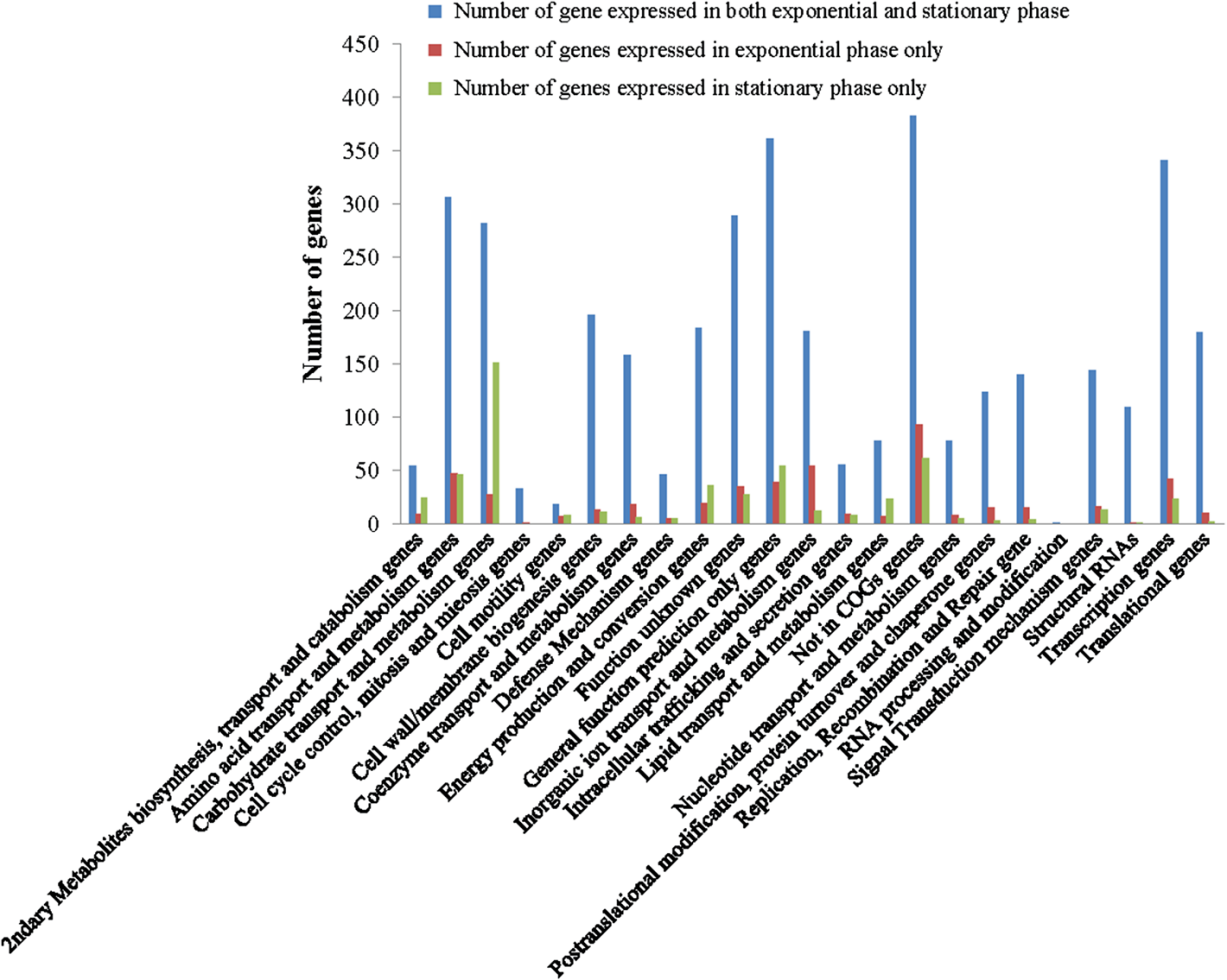
Categorization of expressed genes by COG function.

From our ChIP-chip data, we detected 2677, 1227, and 1066 binding sites for RNAP, RpoD, and RpoS, respectively. The RNAP-guided transcript segmentation method integrates the presence or absence of a particular transcript with RNAP-binding information, which minimizes the error associated with the assembly of unrelated transcripts [24]. This methodology has been applied to this study. Gene expression data supported by RNAP binding site data suggest that there are 3660 RNAP-guided transcript segments (RTSs) in *K. pneumoniae* MGH 78578. Among this group, 1979 RTSs were detected in both the exponential and stationary phase while 678 and 1003 RTSs were detected only during exponential phase and stationary phase growth, respectively (Additional file 1). The 3660 RTSs include the expression of 4752 annotated genes out of a total of 5315 genes in the current genome annotation (89.4%). Based on this annotation, the expression of 4222 and 4299 genes were detected during exponential and stationary phase, respectively, of which 3769 genes were expressed during both growth phases. Thus, there were 453 and 530 genes that were expressed during exponential and stationary phase only, respectively. Interestingly, a large number (151) of the 530 genes that were expressed only during stationary phase growth play a putative role in carbohydrate transport and metabolism based on their COG classification (Figure 2). The functions of these 151 genes are enriched for sugar transport, sugar isomerization, glycoside hydrolysis, and phosphotransferase system (PTS), which are all related to carbohydrate uptake [28].

To establish transcription start sites, we performed a simultaneous analysis of both our raw TSS data and the 3660 RTSs and assigned a TSS if it appeared within 200 bp from the 5^′^ start point of an RTS. This analysis resulted in a total of 3585 TSS signals, 3322 on the chromosome and 263 on the five plasmids. One-hundred ninety-three RTSs were observed to have two or more TSSs. Based on COG classification, the largest group among these 193 RTSs was a set of 26 that are involved in transcription (Additional file 2). Four of these 26 transcription-related RTSs have three or more TSSs. For example, the RTS that includes KPN_01305, a transcriptional regulator involved in biosynthesis and transport of aromatic amino acids, has four TSSs. The existence of multiple TSSs suggests that this and other similar genes are transcribed under multiple, specific conditions rather than under conditions of general growth. When the TSS data was extended to include both the RTS and expression profiling data, we observed 83 leaderless mRNAs, defined here as transcripts with a 5^′^ UTR length of less than or equal to 5 bp. This number is nearly double what has been reported for other bacteria such as *Salmonella enterica* serovar Typhimurium strain SL1344 (23 transcripts) [27], *Helicobacter pylori* (34 transcripts) [26,29], and *Geobacter sulfurreducens* (52 transcripts) [25].

### Non-coding genes

Among non-coding RNAs, the current annotation for the *K. pneumoniae* MGH 78578 genome contains 86 tRNAs and 25 rRNAs and an unknown number of small RNAs (sRNA). The presence of sRNAs are much more difficult to predict because genome annotation algorithms are based predominantly on protein coding regions. The possible existence and location of sRNAs are therefore frequently extracted from whole-genome RNA expression data sets that probe not just open reading frames, but the intergenic regions where sRNAs are located as well [30,31].

Since our tiled-array data provide this whole-genome information, we examined whether we could detect sRNAs in *K. pneumoniae* by analyzing unannotated transcripts in our data set using the Rfam database [32]. Out of 447 unannotated transcripts, 15 of them matched an sRNA already reported in Rfam (Additional file 3). Among this list, we could detect high-level transcription of SraD (genomic position: 3318095~3318169, + strand), SroB (genomic position: 510363~510437, + strand) and CsrC (genomic position: 4572620~4572846, + strand) during stationary phase growth only, an observation that is consistent with data from other Enterobacteria [33-36]. Nine of the sRNAs, RyhB, SraL, SroB, SraC/RyeA, MicF, SraD, GcvB, SraH and IsrN, are reported to possess the ability to bind Hfq, a bacterial RNA binding protein [37]. Six sRNAs, SroB, SraC/RyeA, MicF, SraD, GcvB, and SraH, are reported to regulate protein translation through antisense binding to target mRNAs. Interestingly, we could detect the transcription of both RyeB and SrcC/RyeA sRNA even though their genomic locations overlap on opposite strands (genomic position of RyeB: 2580980~2581070, – strand; genomic position of SrcC/RyeA: 2580976~2581120, + strand). However, we could detect transcription of SrcC/RyeA only during exponential phase and RyeB only during stationary phase. This observation suggests that the two sRNAs might act in a coordinated manner to regulate different aspects of growth rather than in a concerted, simultaneous manner. We cannot rule out the possibility, however, that SrcC/RyeA and RyeB might be expressed at low levels during stationary and exponential phase, respectively, that are below the detection limits of the measurement and data analysis systems employed here.

### Putative open reading frames

Several RTSs in our data sets did not correspond to any ORFs in the current sequence-based annotation for *K. pneumoniae* MGH 78578. For each of these unannotated transcripts, we investigated whether they might show homology to any known gene products by first searching for start and stop codons in the RTS that yielded the longest DNA sequence and had the same reading frame. This DNA sequence was then translated into a peptide sequence and a BLASTp search performed against the RefSeq database with a cutoff E-value of 0.001. This analysis resulted in 119 putative ORFs, 40 of which reside on one of the five plasmids harbored by this strain (Additional file 4). Most of these putative ORFs are currently annotated as hypothetical proteins, but several show high homology (< E-110, Additional file 4) to annotated genes from other strains or species of *Klebsiella* or to members from other genera. In turn, this close match raises the probability that the underlying putative ORF does indeed encode a known gene product. For example, the sequence for ‘RTS_NC_009648_322945_323714_+_exp’ is homologous to HAD hydrolase from *Klebsiella* sp. MS 92–3, but is not annotated as such in *K. pneumoniae* MGH 78578. Other examples include a set of seven RTSs encoded by the pKPN5 plasmid that show homology to resolvase proteins from *E. coli* MS 107–1 (Additional file 4). These and other examples (Additional file 4) highlight the significant strength that comes from the use of sample-matched multi –omic data sets to experimentally refine genome annotations based primarily on computational predictions.

### Antisense transcripts

Through further analysis of unannotated transcripts, we identified 185 probable antisense transcripts in the *K. pneumoniae* transcriptome using a cutoff of 90% overlap to the corresponding gene (Additional file 5). This number decreases to 146 probable antisense transcripts when a cutoff of 100% overlap is used. We used the classification scheme of Yin et al. [38] to group the antisense transcripts into three categories: 5^′^ overlapping, 3^′^ overlapping, and ‘completely covered’. This categorization indicates which parts of the two sequences overlap [38]. We further divided ‘completely covered’ into two sub-categories: ‘antisense RTS completely covered by sense RTS’ and ‘sense RTS completely covered by antisense RTS’. The current annotation of the *K. pneumoniae* MGH 78578 genome lists 5305 genes, approximately 800 more than *E. coli* or *Bacillus subtilis*; therefore, 3.5% of the genes in *K. pneumoniae* have antisense transcription using an overlap of 90%, which is a similar value to what has been reported for *E. coli*[24]. Additional studies in *E. coli*[39] as well as data from other bacteria [40,41] suggest, however, that the proportion of antisense transcripts is closer to ~10-20% of the number of genes in a bacterium. Consequently, the smaller number reported here for *K. pneumoniae* might reflect an incomplete list of antisense transcripts due to low detection sensitivity.

The expression of antisense transcripts for certain genes often varied with the growth phase. For example, we detected antisense transcription from 8 tRNA genes during stationary phase but only one of these genes, the tRNA for serine (KPN_02431), had antisense transcription during exponential phase. Similarly, there was antisense transcription for fructose-1,6-bisphosphatase (KPN_04626), an amino acid exporter (KPN_02015), the transcriptional regulator gene *lysR* (KPN_02148), and the transcriptional regulator gene *argR* (KPN_03645) during stationary phase but not exponential phase. LysR is negatively autoregulated and coordinately activates transcription of *lysA* (KPN_03252), which encodes the enzyme catalyzing the last step in lysine biosynthesis [42,43]. ArgR complexed with L-arginine represses the transcription of several genes involved in the biosynthesis and transport of arginine and histidine, and activates genes for arginine catabolism [44,45]. ArgR represses the expression of ABC transporters for putrescine, lysine, and ornithine as well [46]. Since the inhibition of this large set of genes leads to the reduced uptake of these nutrients, the regulation of ArgR expression by antisense transcription is one possible way to adjust metabolism during stationary phase.

We detected antisense transcripts for the *marR* and *marB* genes within the *marRAB* operon during exponential phase growth. We focused attention on this operon since MarA is known to play a role in pathogenesis: the protein activates genes that mitigate the effects of exposure to environmental stresses such as antibiotics and oxidants [47,48]. MarR is the transcriptional repressor of the *marRAB* operon, but the function of MarB is unknown. We could not detect any antisense transcription for *marA*, suggesting that the regulation of this transcriptional activator occurs through MarR and possibly MarB rather than antisense control of *marA* itself. Curiously, we detected antisense transcripts for *soxS*, which is a dual transcriptional activator that helps to protect the cell against oxidative stress [49], despite the absence of antibiotics or other factors known to promote this phenomenon. Many factors contribute to the ability of *K. pneumoniae* to resist many antibiotics, but these observations suggest that transcriptional regulators and their antisense transcripts might play a role in this process.

### Transcription network among sigma factors

**Figure 3:**
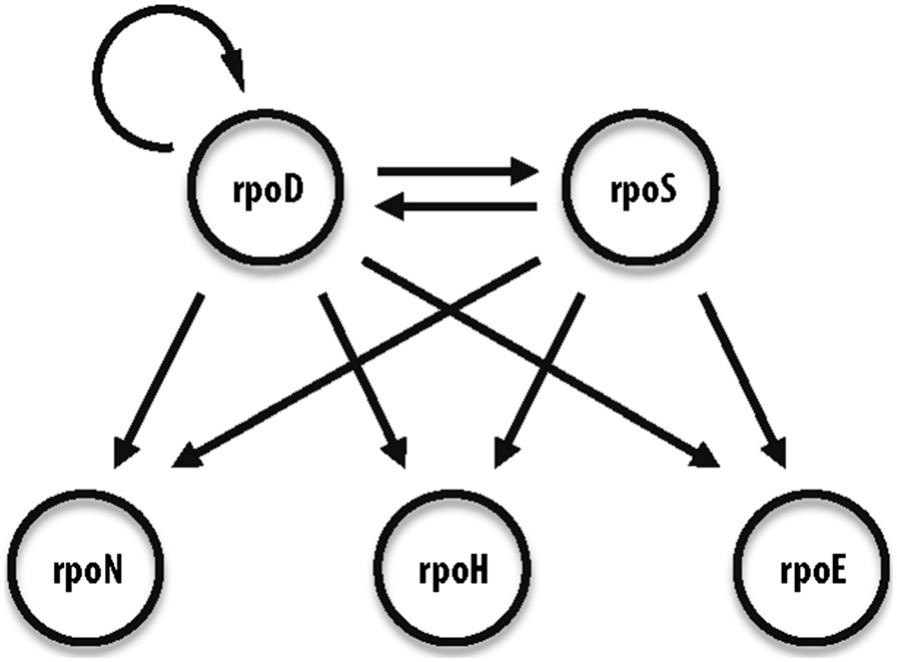
**Interaction network of the five sigma factors in *K. pneumoniae *based on RpoD and RpoS chIP-chip binding data.**

According to the current annotation, *K. pneumoniae* has five major sigma factors – RpoD, RpoS, RpoN, RpoH, and RpoE. Because RpoD and RpoS are the major sigma factors that are active in exponential phase and stationary phase, respectively [50,51], we performed chIP-chip experiments to determine the binding sites for RpoD during exponential phase and RpoS during stationary phase. We found that RpoD could bind upstream of the genes that encode each of the five sigma factors, including its own (Figure 3), data which are consistent with observations from *E. coli*[52]. In contrast, RpoS binding sites were detected throughout 5^′^ upstream regions for each sigma factor gene except its own (Figure 3). RpoS has been shown to bind to the promoter of *rpoH* in *E. coli*[53], but the observation that RpoS can bind to and regulate the expression of *rpoD, rpoN,* and *rpoE* as well in a member of the Enterobacteriaceae appears to be a novel finding.

When phase-specific expression levels of the five sigma factors were compared, *rpoD*, *rpoN*, and *rpoH* had higher expression levels in exponential phase than in stationary phase (Additional file 6), whereas *rpoS* and *rpoE* had higher expression levels in stationary phase. The expression level of *rpoD*, *rpoN*, and *rpoH* in stationary phase decreased to 48%, 47%, and 73%, respectively, when compared to those in exponential phase. In contrast, the expression level of *rpoS* and *rpoE* in stationary phase increased to 430% and 497%, respectively. The dramatic increase of expression level of *rpoE* is in accordance with a previous report [54], and implies that RpoE plays a pivotal role in cell survival during prolonged stationary phase.

### RpoD and RpoS DNA binding motifs

**Figure 4:**
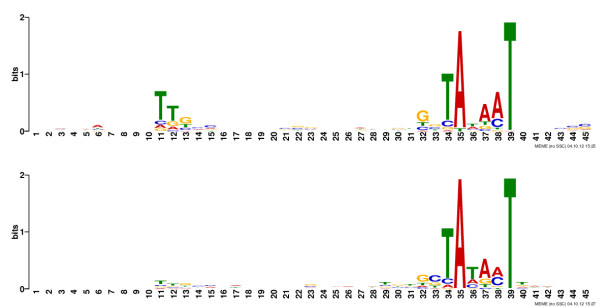
**DNA promoter binding motifs for RpoD (top) and RpoS (bottom) in the *K. pneumoniae *genome.**

The −10 and −35 sequence motifs for RpoD and RpoS promoter binding sites in *K. pneumoniae* extracted from our RpoD and RpoS chIP-chip and TSS data sets are identical to those found in *E. coli*, likely reflecting high conservation of these two sigma factors (the amino acid sequence similarity is 95.9% for RpoD and 98.5% for RpoS). There is a strong TAtaaT signal (lower-case characters indicate an information content <1 bit) at the −10 position in promoters recognized by RpoD (Figure 4), which exactly matches that found in *E. coli*[55] and *Salmonella enterica* serovar Typhimurium [27]. Similarly, a TTgaca consensus signal was found at the −35 position that closely matches that found in *E. coli*. For RpoS, our data suggest that its binding motif in *K. pneumoniae* at the −10 position is TAta(a/c)T (Figure 4), whereas the same element in E. coli is TAYaCT (Y denotes T or C) [55]. Immediately upstream of this motif in *K. pneumoniae* is a gc sequence, and immediately downstream is a taa sequence, both of which are also characteristic features of *E. coli* RpoS −10 promoter elements.

### Transcription of putative virulence genes

The *K. pneumoniae* MGH 78578 genome contains a number of genes that encode putative virulence factors such as capsular polysaccharides (CPS), siderophore biosynthesis and transport, LPS biosynthesis and transport, and fimbriae [56]. In *K. pneumoniae*, the expression levels of these genes during stationary phase growth decreased to less than half their exponential phase values (Additional file 6).

The expression of genes associated with siderophore biosynthesis during stationary phase dropped to ~35% of their corresponding exponential phase level (Additional file 6). Reinforcing the expression data, we detected 20 RTSs during exponential phase that contained one or more genes related to siderophore biosynthesis, but we could detect only 10 such RTSs during stationary phase (Additional file 7). When the absolute signal intensity of siderophore-associated genes is taken into account (average log_2_(signal) = 5.92) and compared to the baseline signal (average log_2_(signal) = ~6), siderophore-associated genes have nearly no expression during stationary phase.

The expression data for genes involved in CPS biosynthesis followed a similar trend as the expression data for siderophore biosynthesis, but the RTS data differed between the two. During stationary phase, the expression levels of CPS-associated genes fell to 10 - 40% of their values during exponential phase. On the other hand, more RTSs containing CPS-associated genes were detected during stationary (26) than exponential (24) phase. These data imply that post-transcriptional regulation might play a greater role in modulating the transcript abundance of CPS-associated genes than siderophore-associated genes during stationary phase.

In contrast to siderophore- and CPS-associated genes, genes associated with both fimbriae and LPS have similar expression levels in both exponential and stationary phases. The average log_2_(signal) for the two were ~6.5 and ~8, respectively (Additional file 6). Interestingly, however, several fimbriae-associated genes such as KPN_00843 (*ompX*, outer membrane protein X) and the gene cluster KPN_03275 ~ KPN_03279 (putative fimbriae-related genes, putative fimbria usher protein and putative pili assembly chaperone) showed much higher expression levels during both growth phases (log_2_(signal) ≥ 12) than other loci that are also associated with fimbriae. Genbank currently does not associate the KPN_03275 ~ KPN_03279 cluster with a specific type of fimbriae, but they are ~99% homologous at the nucleotide level to the *mrk*JFDCB cluster from *K. pneumoniae* NTUH-2044 that encodes type 3 fimbriae. This observation implies that type 3 fimbriae constitute the major class of fimbria expressed by *K. pneumoniae* MGH 78578. In contrast to fimbriae, little variation was detected among the full set of genes associated with LPS biosynthesis, implying that LPS is continually synthesized regardless of growth phase.

## Discussion

Genome sequences are most commonly annotated using bioinformatics-based algorithms, but these algorithms can misannotate genes or introduce other errors [57]. Moreover, a genome sequence by itself provides scant information concerning its functional operation, for example which genes are activated or repressed under specific growth conditions and how their expression is regulated. Against this backdrop, experiment-based techniques such as gene expression and other -omics data provide a foundation with which to verify or correct computation-based annotations at a genome-wide level. We report here such data for *Klebsiella pneumoniae* MGH 78578 through the integrated analysis of gene expression, ChIP-chip of RNAP, RpoD and RpoS, and TSS data during exponential and stationary phase growth. The integrated analysis of these different data sets ensures that findings from one particular data set are reinforced by another, thereby minimizing potential false-positive and -negative findings that might emerge when these data sets are analyzed in isolation [58,59].

Small RNAs are increasingly recognized as important, ubiquitous elements that regulate mRNA half-life, protein translation, and other processes, thereby providing an additional layer of regulatory control of multiple target genes [60]. We detected 15 sRNAs in *K. pneumoniae* through comparison of intergenic transcripts in our data sets to known sRNAs in the Rfam database. Nine of these fifteen have been reported to act through Hfq, and the expression levels for six of them changed at least two-fold in a Δ*hfq* knockout mutant of *K. pneumoniae*[61]*.* The expression level for a seventh, RyhB, changed by less than two-fold in the mutant, while an additional two Hfq-binding sRNAs, SraL and IsrN, are newly detected in our data set. All fifteen sRNAs detected here have also been detected in both *E. coli* and *S.* Typhimurium [27,62], suggesting the existence of a common regulatory network involving these sRNAs that is shared among multiple Enterobacteria. *K. pneumoniae* likely contains many more sRNAs than the 15 detected here, however, since greater numbers of sRNAs have been reported for both *E. coli* and *S.* Typhimurium [27,62].

As with sRNAs, we detected a much smaller number of TSSs in *K. pneumoniae* than has been reported for *E. coli* (3585 in *K. pneumoniae* versus 4133 in *E. coli*[24]) and transcription units (3660 in *K. pneumoniae* versus approximately 4661 in *E. coli*[24]) even though the current GenBank annotations list approximately 800 more genes for *K. pneumoniae* than for *E. coli*. These differences likely stem from the greater number of growth conditions that were investigated in the *E. coli* study, an observation that highlights the plasticity of the transcriptional architecture within these two bacteria as they respond to different environments. *S.* Typhimurium by comparison has been reported to contain much fewer TSSs, approximately 1900 in number [27], but this disparity likely arises from the different data analysis procedures that were employed in the *S.* Typhimurium study versus those for *K. pneumoniae* and *E. coli*.

In contrast to sRNA and TSS data, we detected a greater number of leaderless RNAs for *K. pneumoniae* than have been identified to date in other bacteria. Although this observation suggests that these transcripts might have a functional impact or evolutionary significance that is unique to *K. pneumoniae*, we anticipate that deep sequencing of the transcriptomes from additional microorganisms under multiple growth conditions will eventually yield a similar amount of leaderless RNAs.

Beyond delineating the transcriptional architecture of *K. pneumoniae*, the data presented here highlight the significant impact that the growth phase can have on the expression of virulence genes and, by extension, on drug target selection. For instance, one possible antibiotic development strategy is to interfere with non-essential microbial processes such as capsule, siderophore, and fimbriae biosynthesis and quorum sensing that result in weakened virulence but do not kill the pathogen outright. Although there is some debate [63,64], such strategies are attractive in large part because resistance is expected to emerge at a much slower rate, thereby prolonging the clinical utility of drugs designed with this mechanism of action. Since these processes are non-essential, however, the underlying enzymes might not be present under all conditions. Without a target to inhibit, antibiotics developed against these enzymes would therefore be expected to have little effect. Our data indicate that attempts to inhibit *K. pneumoniae* enzymes involved in siderophore biosynthesis might fall under this scenario since the transcriptomic and RTS signals from genes involved in this process are much lower during stationary phase than during exponential phase. These findings emphasize the need for in-depth studies to validate targets before the start of an antibiotic discovery program, in particular to establish whether a potential target enzyme is ultimately produced under infection-relevant conditions.

## Conclusions

In conclusion, we report here the operational annotation of the *Klebsiella pneumoniae* MGH 78578 genome during exponential and stationary phase growth in glucose M9. We identified numerous RTSs, unannotated transcripts (i.e., transcription from intergenic regions), different types of regulatory RNAs, and putative ORFs. Additional experimental data to confirm the existence of sRNAs, antisense transcripts, and putative ORFs would yield further insight into important mechanisms underlying transcriptional regulation of this important human pathogen.

## Methods

### Bacterial strain, medium and growth condition

Glucose M9 minimal media was used as the primary culture medium. Glucose (2 g/L) M9 minimal media is composed of 2 mL/L of 1 M MgSO_4_, 100 μL/L of 1 M CaCl_2_, 12.8 g/L Na_2_HPO_4_·7H_2_O, 3 g/L KH_2_PO_4_, 0.5 g/L NaCl, 1 g/L NH_4_Cl and 1 ml trace element solution (100X) containing 1 g EDTA, 29 mg ZnSO_4_·7H_2_O, 198 mg MnCl_2_·4H_2_O, 254 mg CoCl_2_·6H_2_O, 13.4 mg CuCl_2_, and 147 mg CaCl_2_. Seed cultures of *K. pneumoniae* were made by inoculating frozen stocks made with 20% glycerol into 3 mL of glucose M9 minimal media and incubating at 37°C. After overnight growth, 5 mL of the seed culture was inoculated into 50 mL fresh glucose M9 minimal media and further cultured at 37°C until it reached an appropriate optical density at 600 nm (OD).

### Gene expression profile analysis

Three milliliters of cell culture media in mid-exponential (OD=0.6) phase or stationary (OD=1.3) phase were mixed with 6 mL RNAprotect Bacteria Reagent (Qiagen, Valencia, CA). Samples were immediately vortexed for 5 seconds and incubated for an additional 5 minutes at room temperature. Samples were then centrifuged at 5000 g for 10 minutes and the supernatant discarded. Total RNA samples were then isolated using a RNeasy Plus Mini kit (Qiagen, Valencia, CA) according to the manufacturer’s instructions. Extracted RNA samples were quantified using a NanoDrop 1000 spectrophotometer and the quality of the isolated RNA was checked by visualization on agarose gels and by measuring the sample’s A_260_/A_280_ ratio (>1.8). Ten μg of total RNA was used to make cDNA with amino-allyl dUTP by reverse transcription. The amino-allyl labeled cDNA samples were then coupled with Cy3 monoreactive dyes (Amersham/GE Healthcare, Pittsburgh, PA). Cy3-labeled cDNAs were digested with DNase I (Epicentre/Illumina, Madison, WI) to generate 50~300 bp fragments. High-density oligonucleotide tiling arrays custom manufactured by Roche NimbleGen that consisted of 379,528 50-mer probes spaced 30 bp apart across the whole *K. pneumoniae* genome were used. Hybridization, wash and scan were performed according to the manufacturer’s instructions. Probe level data were normalized with the RMA (robust multi-array analysis) algorithm in NimbleScan 2.4 without background correction

### ChIP-chip experiment

A previously reported ChIP-chip protocol [65,66] was adopted here for *K. pneumoniae*. Genome-wide RNAP (Additional file 8) and RpoD (Additional file 9) binding sites were identified using cultures grown to mid-log phase in triplicate. Corresponding RpoS binding site identification was carried out using cultures grown to stationary phase, also in triplicate (Additional file 10). Six μL each of RNAP, RpoD, and RpoS antibody (all from Neoclone, Madison, WI) were used for each experiment. As a control (mock-IP), 2 μg of normal mouse IgG antibody (Upstate/Millipore/Merck, Billerica, MA) was used. Real-time quantitative PCR was performed with previously known binding sites to test the enrichment of the immunoprecipitated (IP) DNA library [66]. qPCR and amplification of DNA was carried out according to the method of Cho et al. [66]. Samples confirmed to be enriched for IP DNA were next hybridized to the microarray, washed, and scanned according to the manufacturer’s directions (Roche NimbleGen).

### TSS identification

Total RNA was extracted from two biological replicates for each growth condition using the same method used to acquire gene expression profiles. Terminator 5^′^-Phosphate Dependent Exonuclease (Epicentre/Illumina, Madison, WI) was used to enrich 5^′^ tri-phosphorylated mRNAs from the total RNA including 5^′^ mono-phosphorylated ribosomal RNA (rRNA) and any degraded mRNA at 30°C for 1 hr following the manufacturer’s instructions. The reaction was terminated by adding 1 μL of 100 mM EDTA (pH 8.0). 5^′^ tri-phosphorylated RNAs were precipitated by standard ethanol precipitation with 40 μg of glycogen. RNA was precipitated at −80°C for 20 min and pelleted, washed with 70% ethanol, dried in a Speed-Vac for 7 minutes without heat, and resuspended in 20 μL nuclease free water. The tri-phosphorylated RNA was then treated with RNA 5^′^-polyphosphatase (Epicentre/Illumina, Madison, WI) at 37°C for 30 minutes to generate 5^′^-end mono-phosphorylated RNA for ligation to adaptors. After the 5^′^-polyphosphatase treatment, RNA was extracted using phenol-chloroform and ethanol precipitation.

To ligate 5^′^ small RNA adaptor (5^′^-GUUCAGAGUUCUACAGUCCGACGAUC-3^′^) to the 5^′^-end of the mono-phosphorylated RNA, the enriched RNA samples were incubated with 100 μM of the adaptor and 2.5 U of T4 RNA ligase (New England BioLabs, Ipswich, MA). cDNAs were synthesized using the adaptor-ligated mRNAs as template using a modified small RNA reverse transcriptase (RT) primer from Illumina (5^′^-CAAGCAGAAGACGGCATACGANNNNNNNNN-3^′^) and Superscript II Reverse Transcriptase (Life Technologies, Carlsbad, CA). The RNA was mixed with 25 μM modified small RNA RT primer and incubated at 70°C for 10 min and then at 25°C for 10 min. Reverse transcription was carried out at 25°C for 10 min, 37°C for 60 min, and 42°C for 60 min, followed by incubation at 70°C for 10 min. After the reaction, RNA was hydrolyzed by adding 20 μL of 1 N NaOH and incubation at 65°C for 30 min. The reaction mixture was neutralized by adding 20 μL of 1 N HCl. The cDNA samples were amplified using a mixture of 1 μL of the cDNA, 10 μL of Phusion HF buffer (New England BioLabs, Ipswich, MA), 1 μL of dNTPs (10 mM), 1 μL SYBR green (Qiagen, Valencia, CA), 0.5 μL of HotStart Phusion DNA polymerase (New England BioLabs, Ipswich, MA), and 5 pmole of small RNA PCR primer mix. The amplification primers used were 5^′^-AATGATACGGCGACCACCGACAGGTTCAGAGTTCTACAGTCCGA-3^′^ and 5^′^-CAAGCAGAAGACGGCATACGA-3^′^. Amplification was monitored by a LightCycler (Bio-Rad) and stopped at the beginning of the saturation point. Amplified DNA was run on a 6% TBE gel (Life Technologies, Carlsbad, CA) by electrophoresis and DNA ranging from 100 to 300 bp were selected. Gel slices were dissolved in two volumes of EB buffer (Qiagen, Valencia, CA) and 1/10 volume of 3 M sodium acetate (pH 5.2). The amplified DNA was ethanol-precipitated and resuspended in 15 μL DNAse-free water. The final samples were then quantified using a NanoDrop 1000 spectrophotometer. The amplified cDNA libraries were sequenced on an Illumina Genome Analyzer. Sequence cDNA libraries for *K. pneumoniae* were aligned onto the reference genome sequence for this organism (Genbank accession number: CP000647.1 to CP000652.1), using Mosaik ( http://code.google.com/p/mosaik-aligner) with the following arguments: hash size = 10, mismatach = 0, and alignment candidate threshold = 30 bp. The two biological replicates were processed separately, and only sequence reads present in both replicates and aligned to unique genomic location were considered for further study. The genome coordinates of the 5^′^-end of these uniquely aligned reads were defined as potential TSS. GenBank lists 5185 ORFs for this organism.

### Prediction of putative open reading frames (pORFs)

As a first step, transcripts from intergenic regions (i.e., unannotated transcripts) were collected. For each unannotated transcript, we searched for start and stop codons that formed the longest transcript and had the same reading frame. This sequence was defined as a putative ORF (pORF) and translated into a protein sequence. Theoretically translated protein sequences were searched against the RefSeq database using BLASTp. Best hits with E-value less than or equal to 0.001 were listed as pORFs.

### Prediction of putative small RNAs (sRNAs)

Each unannotated transcript was searched against the Rfam database ( http://rfam.sanger.ac.uk/). sRNA search results from Rfam gave homologous sRNA class with E-value, which are listed in Additional file 3. If the unannotated transcript did not match an entry in Rfam, it was assumed not to be an sRNA.

### Prediction of antisense transcripts

Unannotated transcripts were analyzed to determine whether they overlapped with any RTSs from our data set or annotated genes. These unannotated transcripts were listed as antisense transcripts if the overlap was over 90% (Additional file 5).

### RpoD and RpoS binding motif analysis

To identify RpoD-specific promoter sequence motifs, we took 50 bp sequences immediately upstream of TSS signals that were located within RpoD ChIP-chip binding regions and analyzed them using the MEME motif search algorithm. The procedure used to determine RpoS-specific promoter sequence motifs was identical except that we analyzed 60 bp genomic sequences rather than 50.

## Misc

Joo-Hyun Seo and Jay Sung-Joong Hong contributed equally to this work

## Competing interests

The authors declare no competing interests.

## Authors’ contributions

JHS and JSJH carried out the experiments, analyzed the data, and drafted the manuscript. DK participated in data analysis. BKC, TWH, SFT, BOP, and PC participated in the design of the study. SFT, BOP, and PC conceived the study and participated in its design and coordination, and helped to draft the manuscript. All authors read and approved the final manuscript.

## Authors’ information

Raw chIP-chip and transcriptomic data files have been deposited into the GEO database with accession numbers GSE35926 and GSE35927.

## Supplementary Material

Additional file 1 List of all RTSs detected during this study. Click here for file

Additional file 2 RTSs containing more than one TSS. Click here for file

Additional file 3 List of putative sRNAs. Click here for file

Additional file 4 List of putative ORFs. Click here for file

Additional file 5 List of antisense transcripts. Click here for file

Additional file 6 Expression level differences between exponential versus stationary phase for the five sigma factors and known virulence genes. Click here for file

Additional file 7 RTS and chIP-chip data for known virulence genes. Click here for file

Additional file 8 List of all RNAP binding sites and their associated RTSs. Click here for file

Additional file 9 List of all RpoD binding sites and their associated RTSs. Click here for file

Additional file 10 List of all RpoS binding sites and their associated RTSs. Click here for file
